# Temozolomide arrests glioma growth and normalizes intratumoral extracellular pH

**DOI:** 10.1038/s41598-017-07609-7

**Published:** 2017-08-11

**Authors:** Jyotsna U. Rao, Daniel Coman, John J. Walsh, Meser M. Ali, Yuegao Huang, Fahmeed Hyder

**Affiliations:** 1 Magnetic Resonance Research Center, Yale University, New Haven, CT USA; 2 Department of Radiology and Biomedical Imaging, Yale University, New Haven, CT USA; 3 Department of Biomedical Engineering, Yale University, New Haven, CT USA; 4 Department of Neurology, Henry Ford Hospital, Detroit, MI USA

## Abstract

Gliomas maintain an acidic extracellular pH (pH_e_), which promotes tumor growth and builds resistance to therapy. Given evidence that acidic pH_e_ beyond the tumor core indicates infiltration, we hypothesized that imaging the intratumoral pH_e_ in relation to the peritumoral pH_e_ can provide a novel readout of therapeutic influence on the tumor microenvironment. We used Biosensor Imaging of Redundant Deviation in Shifts (BIRDS), which utilizes chemical shifts of non-exchangeable protons from macrocyclic chelates (e.g., DOTP^8−^) complexed with paramagnetic thulium (Tm^3+^), to generate pH_e_ maps in rat brains bearing U251 tumors. Following TmDOTP^5−^ infusion, T_2_-weighted MRI provided delineation of the tumor boundary and BIRDS was used to image the pH_e_ gradient between intratumoral and peritumoral regions (ΔpH_e_) in both untreated and temozolomide treated (40 mg/kg) rats bearing U251 tumors. Treated rats had reduced tumor volume (p < 0.01), reduced proliferation (Ki-67 staining; p < 0.03) and apoptosis induction (cleaved Caspase-3 staining; p < 0.001) when compared to untreated rats. The ΔpH_e_ was significantly higher in untreated compared to treated rats (p < 0.002), suggesting that temozolomide, which induces apoptosis and hinders proliferation, also normalizes intratumoral pH_e_. Thus, BIRDS can be used to map the ΔpH_e_ in gliomas and provide a physiological readout of the therapeutic response on the tumor microenvironment.

## Introduction

Gliomas account for more than 80% of all malignant brain tumors with most patients progressing to highly malignant grade IV glioblastomas (GBMs﻿). Patients with GBMs have a median survival of ∼12 months with only 3–5% of patients surviving for more than 3 years^1^. Surgical resection and radiation therapy, together with adjuvant chemotherapy (e.g., temozolomide (TMZ)), is currently used to treat GBMs clinically. Although TMZ prolongs survival, chemoresistance and recurrence are common^2^. Thus, having reliable markers and methods to assess therapeutic response is of extreme importance for seeking alternative treatment routes. In this context, imaging extracellular pH (pH_e_) has gained importance. A shared trait among cancers is the metabolic shift from oxidative phosphorylation to glycolysis (Warburg effect), which leads to increased acidification of the extracellular milieu as tumor cells extrude H^+^ and lactate produced as a result of increased glycolysis^3^. In response to DNA alkylating agents like TMZ, apoptosis of tumor cells is induced. Consequently a reduction ﻿in tumor burden and glycolytic output is expected, which can be reflected as increased intratumoral pH_e_.

Biosensor Imaging of Redundant Deviation in Shifts (BIRDS) is a 3D chemical shift imaging (CSI) platform where paramagnetically-shifted non-exchangeable protons on (-DOTA) based macrocyclic complexes are directly detected. The proton shifts provide a readout of the physicochemical environment and the signal does not depend on diffusion or blood flow^4–6^. Here we use BIRDS, which is an attractive alternative MR method for molecular imaging, to evaluate the therapeutic efficacy of TMZ by measuring the pH_e_ inside and outside the tumor boundary of U251 gliomas.

## Results

### Effect of TMZ on tumor size, apoptosis, and proliferation

**Figure 1:**
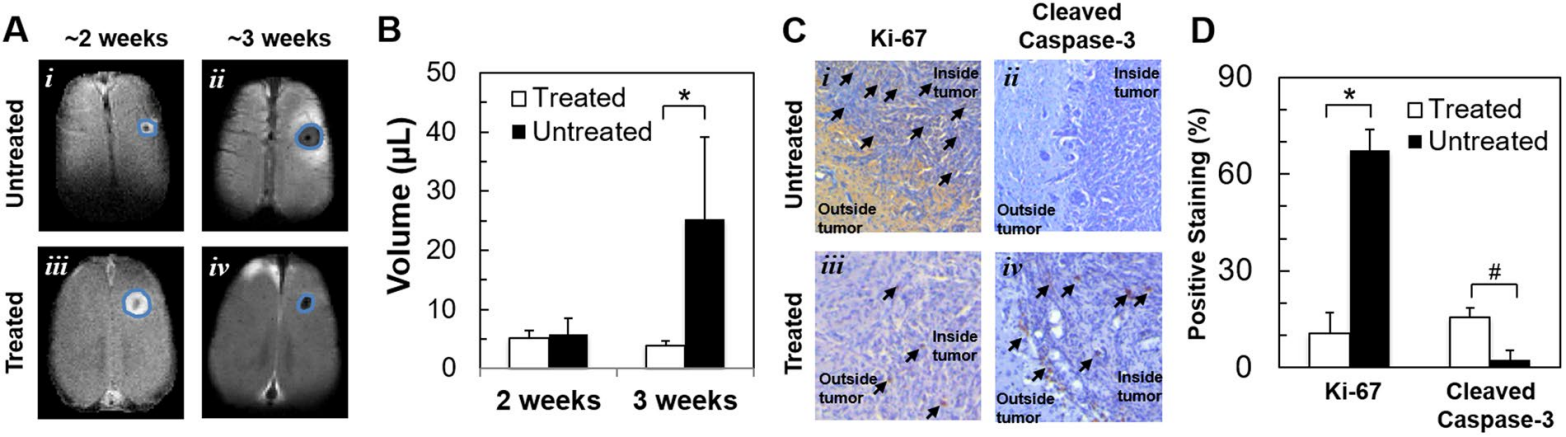
Effect of TMZ treatment on U251 tumor morphology, apoptosis, and proliferation. (**A**) T_1_- and T_2_-weighted MRI at ~2 and ~3 weeks post tumor implantation, respectively, depicting tumor sizes in untreated and TMZ treated U251 tumor bearing rats. ﻿﻿The measurements at ~2 weeks and ~3 weeks were, respectively, made by T_1_ and T_2_ MRI contrast enhacement.﻿﻿ In the treated group, the rats were imaged at 12.6 ± 0.5 and 22.8 ± 0.7 days, whereas in the untreated group the rats were imaged at 12.4 ± 2.2 and 22.0 ± 3.3 days. (**B**) Tumor volume in treated and untreated U251 bearing rats at ~2 and ~3 weeks post tumor implantation, where the difference between treated and untreated groups were significant at later stages (*p = 0.01). (**C**) Ki-67 and cleaved caspase-3 staining in untreated and TMZ treated U251 tumor bearing rats. Arrows point to brown colored positive DAB staining. (**D**) Percentage of cells showing positive staining for Ki-67 and Cleaved Caspase-3. In treated vs. untreated tumors, Ki-67 shows a significant decrease in proliferative index (*p = 0.025) and cleaved caspase-3 shows a significant increase in apoptotic index (#p = 0.001).

﻿Tumor size was ﻿measured﻿ by MRI contrasts generated from water proton longitudinal (T_1_) and transverse (T_2_) relaxation enhancements. ﻿The effect of TMZ on U251 tumor growth was assessed by measuring the tumor volume at ~2 weeks (i.e., 12–14 days using T_1_-enhanced contrast by Gadobutrol) and ~3 weeks (i.e., 22–24 days using T_2_-enhanced contrast by TmDOTP^5−^) post tumor inoculation. Recent experiments^7^ indicate that tumor volumes measured with a T_1_ agent (e.g. Gadobutrol) are nearly identical with those measured using a T_2_ agent (e.g. TmDOTP^5−^) in the same rat. Ambiguity exists in delineating tumors in clinical images with ill-defined tumor boundaries. However, U251 tumors in rodents have a well defined tumor mass with fairly well defined boundaries. Thus, ambiguity in tumor boundaries has minimal effect on the tumor volume measurements compared to clinical scans. The volume of untreated tumors at 2 weeks post tumor inoculation was 5.9 ± 2.7 μL, which increased to 25.3 ± 13.9 μL at 3 weeks. Treated tumors had similar volumes at 2 weeks (5.2 ± 1.2 μL) and 3 weeks (3.9 ± 0.9 μL) (Fig. 1A and B). Thus, tumor sizes were significantly different in treated vs. untreated animals at later stages of tumor growth (p < 0.01) and there was no further tumor growth with therapy.

Expression of Ki-67 and cleaved Caspase-3, shown in Fig. 1C, Supplementary Figure 1 and Fig. 1D respectively, was investigated in treated and untreated rats. Quantitative analysis revealed that the average Ki-67 labeling before TMZ treatment was 67.5%, which reduced to 10.5% following TMZ treatment (p < 0.03) indicative of reduced proliferation. Cleaved Caspase-3 staining before TMZ treatment was 2.5%, which increased to 15.5% following TMZ treatment (p < 0.001) suggesting apoptosis initiation. Expression of MCT-4 was analyzed in a representative sample of untreated and treated tumors and high MCT-4 expression was observed in untreated tumors (Supplementary Figure 2).

### Effect of TMZ on intratumoral and peritumoral acidity

**Figure 2:**
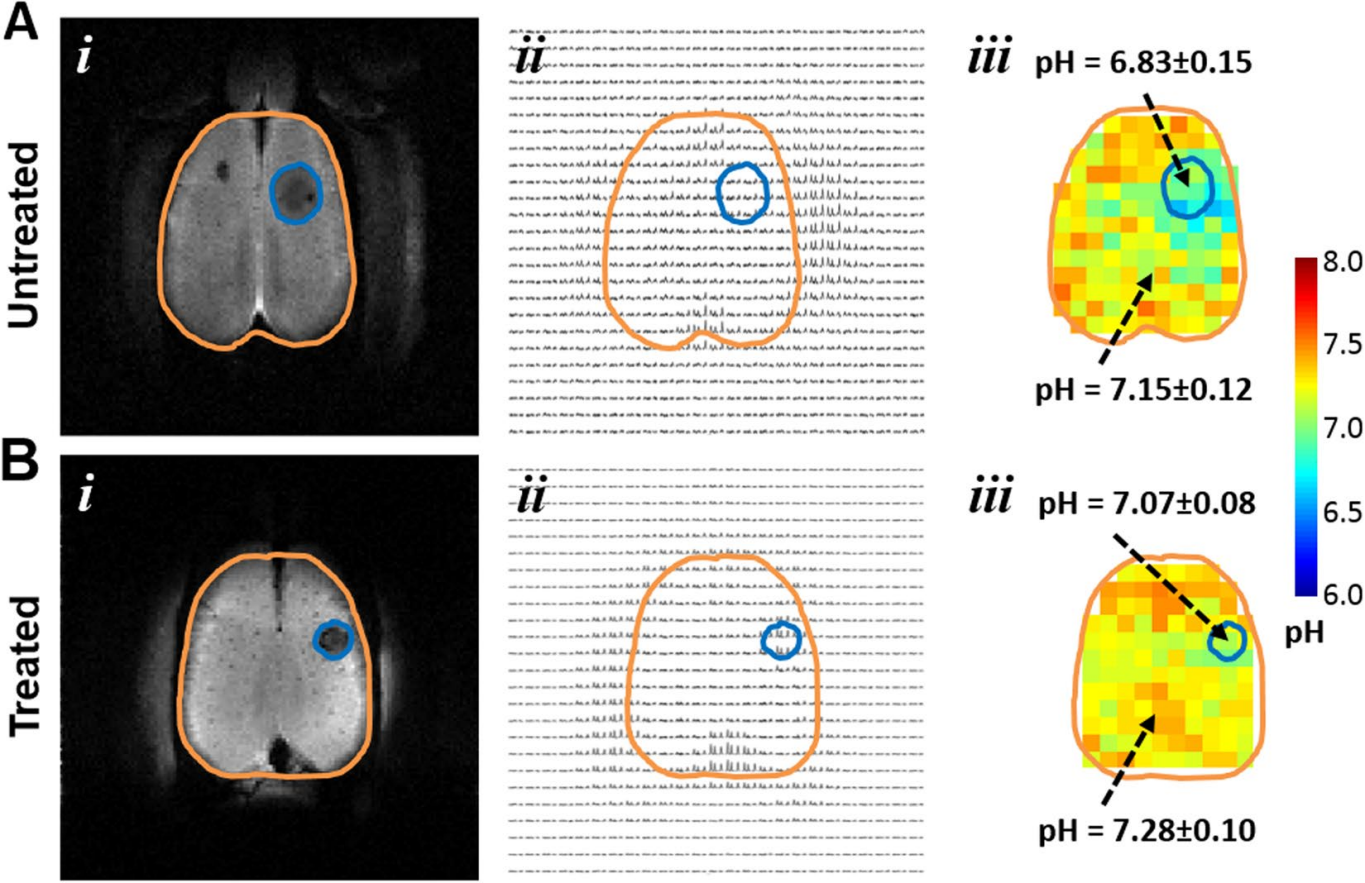
Representative pH_e_ maps from BIRDS in untreated **(A)** and TMZ treated **(B)** rats bearing U251 tumors. **(i)** The T_2_-weighted images identify the tumor boundary (tumor = blue outline; brain = orange outline). **(ii)** CSI data for corresponding slice in (i) shows varying TmDOTP^5−^ levels throughout the brain. **(iii)** Quantitative pH_e_ maps were obtained using multiple TmDOTP^5−^ peaks and the intratumoral and peritumoral pH_e_ average values and their standard deviations (SDs) are indicated. In untreated rats, the tumor is larger and the intratumoral pH_e_ is acidic and spreads beyond the tumor boundary. In treated rats, the tumor is smaller and the intratumoral pH_e_ is near neutral with lower pH_e_ localized within the tumor boundary.

Representative BIRDS data from untreated and treated rats are shown in Fig. 2. The tumors were localized by T_2_ contrast as a result of TmDOTP^5−^ extravasation from immature tumor vasculature^5, 8^. Treated tumors appeared smaller in size compared to untreated tumors (Fig. 2A(i) and B(i); see also Fig. 1A and B). The proton resonances of TmDOTP^5−^ in the corresponding imaging slice are shown in Fig. 2A(ii) and B(ii). As the permeability and clearance rates of TmDOTP^5−^ vary in different tissues, the peak intensities vary across the different regions in the image. As reported previously^5^, BIRDS can determine pH_e_ maps for tumors of different sizes, and both the intratumoral pH_e_ (inside tumor boundary) and peritumoral pH_e_ (outside tumor boundary) can be measured simultaneously. The intratumoral pH_e_ values for the untreated and treated tumors were ~6.8 and ~7.1, respectively (Fig. 2A(iii) and B(iii)). However, the peritumoral pH_e_ values quite distal from the tumor boundary for the untreated and treated tumors were approaching neutral values (i.e., ~7.2 and ~7.3, respectively, in Fig. 2A(iii) and B(iii)). It should be noted, however, that acidic peritumoral pH_e_ values were observed immediately adjacent to the tumor boundary in untreated tumors. This is supported by greater Ki-67 expression in untreated rats when compared to treated rats (Fig. 1C and D). This trend of acidic peritumoral pH_e_ immediately adjacent to the tumor boundary has been observed for other aggressive gliomas^5, 8^.

Because the spatial resolution of pH_e_ maps was lower than that of MR images, we took precautions to report the intratumoral and peritumoral pH_e_ values accurately. In small tumors, where many voxels intersect the tumor boundary defined by MRI, partial contribution from the non-tumor area can confound the results. Thus, voxels that contained more than 50% tumor tissue were considered tumor voxels resulting in at least 2 tumor voxels in the smallest tumors detected.

**Figure 3:**
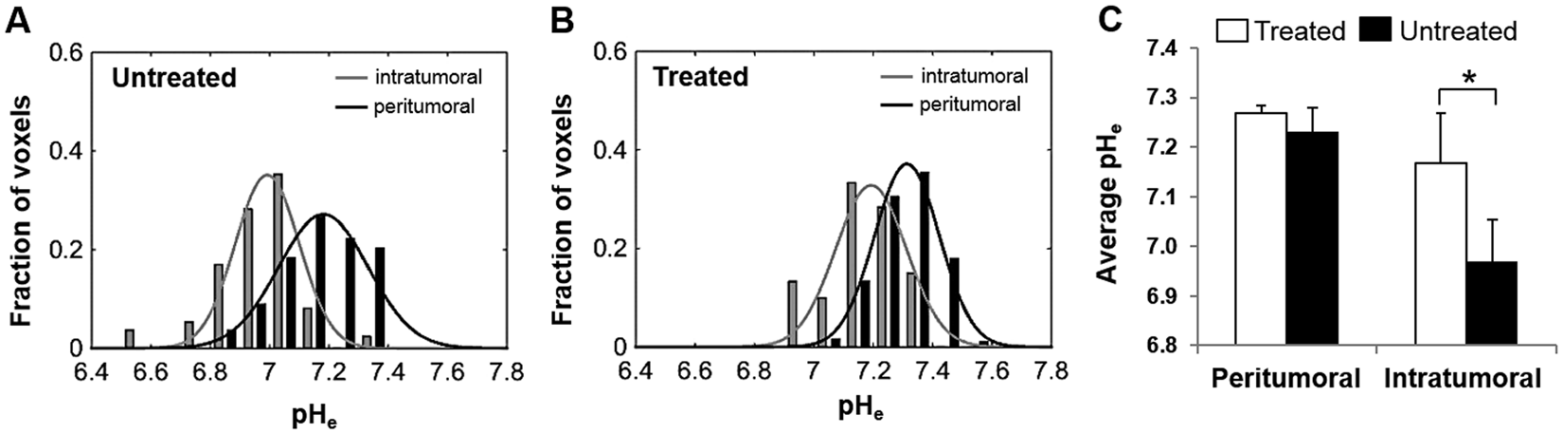
Distribution of pH_e_ values in intratumoral (gray bars) and peritumoral (black bars) regions for untreated **(A)** and TMZ treated **(B)** U251 tumors. Average pH_e_ values (±SD) for intratumoral and peritumoral regions in untreated and TMZ treated U251 tumors **(C)** show that the average pH_e_ value for intratumoral voxels is significantly lower in untreated tumors compared to treated tumors (*p = 0.001).

The intratumoral and peritumoral pH_e_ values for untreated and treated tumors are represented in histograms in Fig. 3A and B, respectively. The histograms were fitted to a Gaussian distribution to obtain the most probable pH_e_ and the full width at half maximum (FWHM) of these histograms. The most probable intratumoral pH_e_ values were higher in treated when compared to untreated tumors. The FWHM values of untreated tumors (~0.4) were higher than treated tumors (~0.3), indicating heterogeneous pH_e_ in untreated rats, which is normalized by TMZ in treated rats. Figure 3C shows the average values of intratumoral and peritumoral pH_e_ in treated and untreated rats. Average intratumoral pH_e_ was significantly (p < 0.01) lower in untreated rats compared to treated rats, whereas in peritumoral regions, no significant difference (p > 0.05) was observed between the two groups.

**Figure 4:**
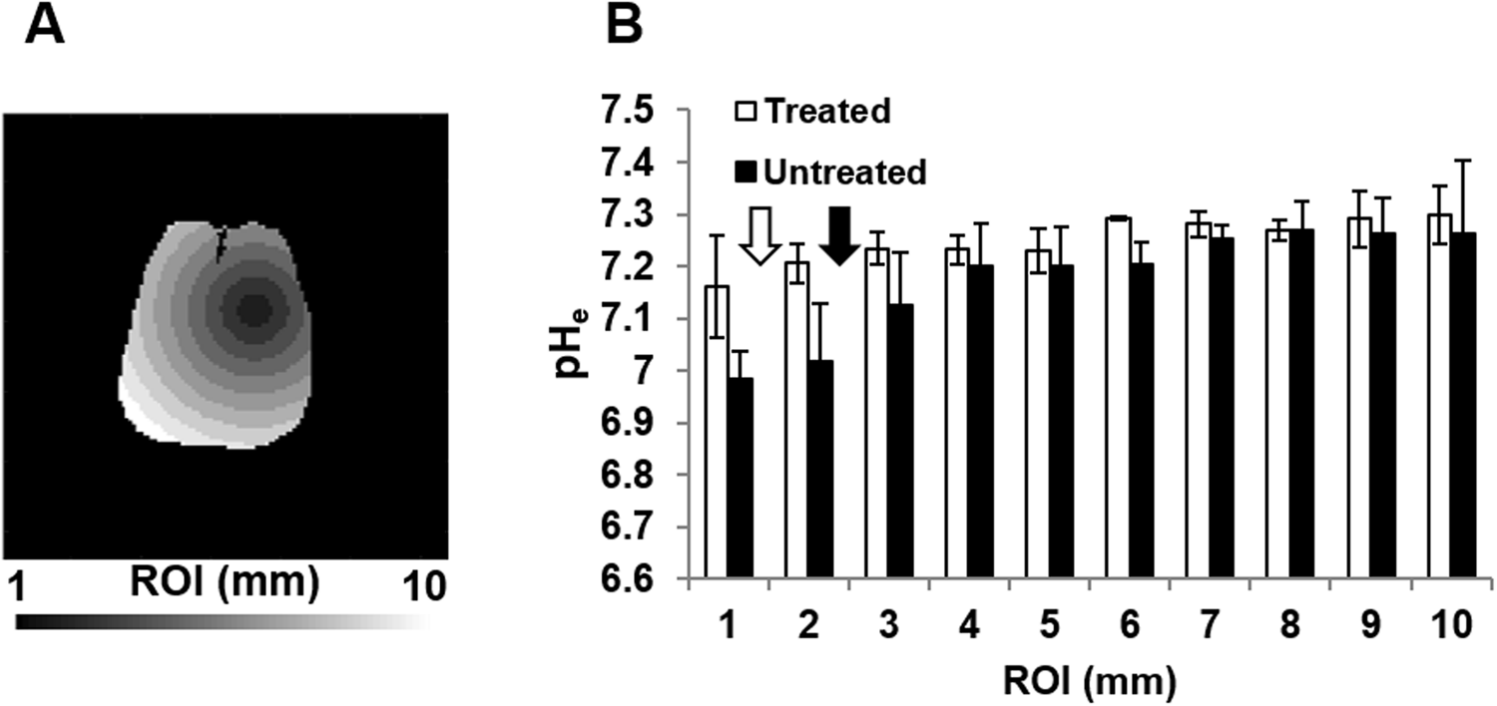
Average pH_e_ values across all animals in ROIs positioned at increasing distances from the tumor center. The T_2_ map for each animal was used to mask the tumor and determine its center of mass. The brain was then segmented into ROIs (**A**) defined by the distance from the corresponding voxel to the center of mass of the tumor. Each ROI was defined as the area bounded by two circles of radius n-1 mm to n mm from the center of mass of the tumor (n = 1 to 10). The average pH_e_ value for each animal was calculated from the pH_e_ values from all voxels inside each ROI. Then, the average pH_e_ values and the corresponding SDs (indicated as the error bar) across animals were calculated for each ROI (**B**). The boundaries between the intratumoral and the peritumoral regions (shown with white and black arrows for the treated and untreated groups, respectively) were calculated from the average tumor volume obtained from the anatomical MR images (Fig. 1B), assuming a spherical tumor shape.

To investigate how pH_e_ is affected in voxels positioned at different distances relative to the center of mass of the tumor, the brain was separated into ﻿﻿regions of interest (ROIs) starting from the center of the tumor and progressing outwards in concentric 1 mm circles (Fig. 4A). The boundaries between the intratumoral and the peritumoral regions (shown with white and black arrows for the treated and untreated groups, respectively, in Fig. 4B) were calculated from the average tumor volume obtained from the anatomical MR images (Fig. 1B), assuming a spherical tumor shape. Lower pH_e_ values were observed in the intratumoral regions (ROIs 1 and 2) in the untreated group (Fig. 4B). In the treated group, the pH_e_ values in the intratumoral regions (ROI 1) were partially normalized by TMZ treatment. The lower pH_e_ value measured in ROI 3 in the untreated group (7.13 ± 0.10) reflects the increased acidity of the tissue surrounding the tumor, which can also be observed in Fig. 2. The treated group, in comparison, has pH_e_ values of the tissue surrounding the tumor (ROI 2) closer to normal values (7.21 ± 0.04). However, the lower average pH_e_ value observed for the untreated group in regions surrounding the tumor (ROI 3) might be due to contributions from larger tumors (with lower pH_e_). For regions distant from the tumor center (ROIs 4 to 10), the pH_e_ values are not significantly different between the treated and the untreated groups (p > 0.05). In addition, heterogeneous pH_e_ distributions in the untreated rats were suggested by larger SDs in each ROI compared to treated rats, except ROI 1. The larger standard deviation for ROI 1 is most likely due to variations in the TMZ effect on intratumoral pH_e_ across different animals. Note that most of the intratumoral regions for the treated group is restricted to ROI 1, based on the tumor boundary estimation described above. The SD for the rest of the ROIs (ROIs 2 to 10) is smaller in the treated group because those ROIs contain mostly normal brain tissue whose pH_e_ is minimally affected by the temozolomide treatment.

## Discussion

Assessment of therapeutic response in GBMs is usually achieved by T_1_- and T_2_-dependent MRI contrasts. However, these methods can be confounded by pseudoprogresson and pseudoresponse. Thus, alternative methods for monitoring therapeutic response are needed.

Various MRS and MRI techniques for pH_e_ mapping have been previously reported. Gallagher *et al*. imaged pH_e_ in mouse lymphoma using hyperpolarized ^13^C bicarbonate measurements^9^. pH_e_-sensitive contrast was measured in C6 glioma rats using GdDOTA-4Amp^5−^, a pH sensitive T_1_ agent^7^. MRI methods based on chemical exchange saturation transfer (CEST), using either diamagnetic or paramagnetic agents have been used to generate pH_e_-sensitive maps such as CEST imaging of the amine protons on glutamine. Amide proton transfer (APT) and amine CEST have been used in both preclinical and clinical settings^10, 11^.

BIRDS is a different technique, which has been used to image the intratumoral-peritumoral pH_e_ gradient in gliomas^5, 8^. Because the molecular readout from the TmDOTP^5−^ protons is chemical shift-dependent, the method is independent of field strength and agent concentration. We reported previously^6^ that the error in pH determination depends on the error in chemical shift measurement, which in turn depends on the signal-to-noise ratio (SNR) for each proton resonance. Typical *in vivo* SNR values for H2, H3 and H6 protons is in the range of 5 to 20, which corresponds to an error in pH measurement in the range of 0.01 to 0.03. While a limitation of BIRDS is the spatial resolution compared to conventional MRI, the pH_e_ readout even at a coarse spatial resolution provides valuable insights into the metabolic state of the tumor microenvironment in relation to its neighboring tissue.

It was observed by Estrella *et al*. (2013) that regions of highest tumor invasion corresponded to regions of lowest pH_e _^12^. So, it is hypothesized that acidic pH_e_ mediates local invasive growth and metastasis. Recently, we showed that extensive acidic pH_e_ in the periphery of the tumor is correlated with increased invasiveness. This is associated with increased presence of Ki-67 positive cells in the tumor boundary of invasive models^5^. Similarly, in untreated tumors we observe lower pH_e_ in the tumor boundary and increased presence of Ki-67 positive cells indicative of increased proliferation and invasive growth. TMZ treatment in U87 rat gliomas has shown alterations in the lactate to pyruvate ratio in comparison with untreated rats due to reduced pyruvate kinase M2 activity^13, 14^. In addition, reduced metabolic output in response to reduced tumor burden suggests altered pH_e_ in intratumoral and peritumoral regions in response to TMZ. We observed an inhibition of tumor growth with TMZ treatment, similar to several previous reports^14–17^. Also in agreement with previous studies, we observed apoptosis induction and reduced proliferation with TMZ treatment^13, 17^.

In a recent report by Huang *et al*.^8^ an alternative method is presented for achieving a transient increase in the circulatory concentration of BIRDS agents (TmDOTP^5−^) by using probenecid. Probenecid is an organic anion transporter inhibitor that is used to decrease the renal excretion rate of antibiotics and certain other drugs and thus achieve increased drug concentration. Co-infusion of probenecid along with TmDOTP^5−^ showed that intratumoral peritumoral pH_e_ gradient was unaffected by co-infusion. This allows longitudinal studies and enables translation of the technique to the clinic.

In conclusion, mapping pH_e_ in intratumoral and peritumoral regions using BIRDS could serve as a biomarker in evaluating response to TMZ therapy in gliomas, which in turn could be potentially applied in evaluating response to a wide range of therapies.

## Materials and Methods

Experiments were performed according to NIH guidelines. Yale University’s animal care and use committee (IACUC) approved the protocol. Scans were conducted on an 11.7 T Agilent (Santa Clara, CA) horizontal-bore spectrometer, with a bore size of 21 cm and maximum gradient strength of 400 mT/m, using a ^1^H surface radio-frequency^18^ (RF) coil (1.4 cm diameter). TmDOTP^5−^ (1,4,7,10-tetraazacyclododecane-1,4,7,10-tetrakis(methylene phosphonate) complexed with thulium) was purchased from Macrocyclics (Dallas, TX, USA), Gadobutrol was obtained from Bayer (Whippany, NJ, USA), TMZ was obtained from Sigma-Aldrich (St. Louis, MO, USA). U251 cells were purchased from American Type Culture Collections (Manassas, VA, USA).

### Preparation and treatment of rats bearing U251 tumors

U251 cells were grown in DMEM low glucose (Gibco) media containing 10% heat inactivated fetal bovine serum with 1% penicillin and streptomycin at 37 °C and 5% CO_2_. Adult, female Athymic nude rats (200–250 g; n = 11), maintained according to approved animal care protocols, were anesthetized with isoflurane (2–3%) and positioned in a stereotaxic instrument. U251 cells were washed and suspended in DMEM low glucose media and injected intrathalamically with a 26 gauge beveled needle into the tip of the right thalamus at coordinates 3 mm to the right from the bregma and 3 mm ventral to the dura. A 5 µL volume of the cell suspension (200,000 cells/µL) was injected in 5 minutes and the needle was left in place for an additional 5 minutes before it was slowly withdrawn. Starting at twelve to fourteen days post injection, TMZ (40 mg/kg) was orally administered daily in 5 rats, in 2 cycles of 4 days each with a gap of 2 days in between the cycles. In both treated (n = 5) and untreated (n = 6) rats, the glioma volume was measured by contrast-enhanced MRI (Fig. 1). At the end of chemotherapy (22–24 days post injection), pH_e_ was measured using BIRDS (Fig. 2).

### Tumor volume and acidity measurements by MRI

Tumor volume was measured with Gadobutrol and TmDOTP^5−^ inducing longitudinal (T_1_) and transverse (T_2_) relaxation enhancements, respectively, by MRI. Spin-echo images with 128 × 128 in-plane resolution, 1 mm slice thickness and field of view (FOV) of 25 × 25 mm^2^, recycle time (TR) of 4 s, and echo time (TE) of 7 ms. These parameters give an in-plane resolution of 0.195 × 0.195 mm^2^ and voxel size of 0.038 mm^3^, i.e., parameters which are sufficient for defining tumors of 1 mm^3^. Although we used T_1_ and T_2_ agents (i.e., Gd^3+^ and Tm^3+^, respectively) in our study to assess tumor sizes, previous work has shown that Gd^3+^ and Dy^3+^ extravasation in the same subject identify the same tumor boundary^7^. The tumor volume was calculated from the difference in the MR image intensity in all MR slices before and after contrast agent (either Gadobutrol or TmDOTP^5−^) injection. The absolute intensity difference before and after contrast agent was divided by the image intensity before contrast agent injection to obtain a relative change in the intensity. The volume of the tumor was assumed to be equal to the volume of the region where the relative intensity change is larger than a threshold value, established by comparison with the relative intensity change measured in the contralateral (left) hemisphere.

At 12–14 days after tumor inoculation (~2 weeks) each rat underwent bolus injection of Gadobutrol (~0.4 mmol/kg) to estimate the tumor volume from increased MRI signal (T_1_ contrast) due to Gadobutrol extravasation. At 22–24 days after tumor inoculation (~3 weeks) each rat underwent infusion of TmDOTP^5−^ (~0.4 mmol/kg) for pH_e_ imaging and the tumor volume was estimated from decreased MRI signal (T_2_ contrast) due to TmDOTP^5−^ extravasation^5^. The anesthetized rats were prepared as described earlier with renal ligation to maintain a high concentration of TmDOTP^5−^ (slowly infused at 0.5–0.7 mL/hr) during BIRDS experiments^6, 19^. Ventilation was adjusted to maintain normal physiology. Body temperature was measured with a rectal probe and no further adjustments in the water-heated pad were made for the entire duration of the experiment (~2 hrs).

Tumor acidity was measured using the BIRDS technique described previously^5^ utilizing 3D CSI with an effective resolution of 1 μL^4^. pH_e_ was calculated using BIRDS as previously described^5^.

### Histopathological study of effect of TMZ therapy

Post experiment, rats were perfuse-fixed with 4% paraformaldehyde and embedded in paraffin. Brain tissue sections (6 µm) were immunohistochemically stained for Ki-67 and cleaved Caspase-3 as previously described^20^. Primary antibody diluted in 1% BSA/PBS was applied overnight at 4 °C for Ki-67 (1:25, Abcam, ab66155) and Caspase-3 (1:25, Cell Signaling Technology, #9661). The sections were incubated with a goat anti-rabbit secondary antibody (1:500, Pierce, #31460) for 60 minutes followed by incubation with metal enhanced 3,3-diaminobenzidine (DAB; Life technologies, #34065) for 10 min. Expression of immunohistochemical markers was quantified by evaluating the presence of DAB staining and visually quantifying positive staining.

### Statistics

All staining, tumor size and pH_e_ results were expressed as mean ± SD and comparisons between groups were assessed by Student’s t-test with two tails where p < 0.05 was considered significant.

### Data availability

The datasets generated during and/or analysed during the current study are available from the corresponding author on reasonable request.

## Electronic supplementary material

Supplementary Figures
