# Supplementary material for: Temozolomide arrests glioma growth and normalizes intratumoral extracellular pH
Source: Sci Rep. 2017 Aug 11;7:7865. doi: 10.1038/s41598-017-07609-7 (PMC5554228; doi:10.1038/s41598-017-07609-7)
Supplement: Supplementary file 1 — Supplementary Figures [file 41598_2017_7609_MOESM1_ESM.pdf]

Temozolomide arrests glioma growth and normalizes intratumoral extracellular pH

Jyotsna U. Rao[*1, 2], Daniel Coman[*1, 2], John J. Walsh[1, 3], Meser M. Ali[4], Yuegao Huang[1, 2], Fahmeed Hyder[*1-3]

1. Magnetic Resonance Research Center, Yale University, New Haven, CT, USA
2. Department of Radiology and Biomedical Imaging, Yale University, New Haven, CT, USA
3. Department of Biomedical Engineering, Yale University, New Haven, CT, USA
4. Department of Neurology, Henry Ford Hospital, Detroit, MI, USA

Address correspondence and reprint requests to:

[*]Jyotsna U. Rao <jyotsna.u.rao@gmail.com> / Daniel Coman <daniel.coman@yale.edu> / D. S. Fahmeed Hyder <fahmeed.hyder@yale.edu>

Supplementary Figure 1

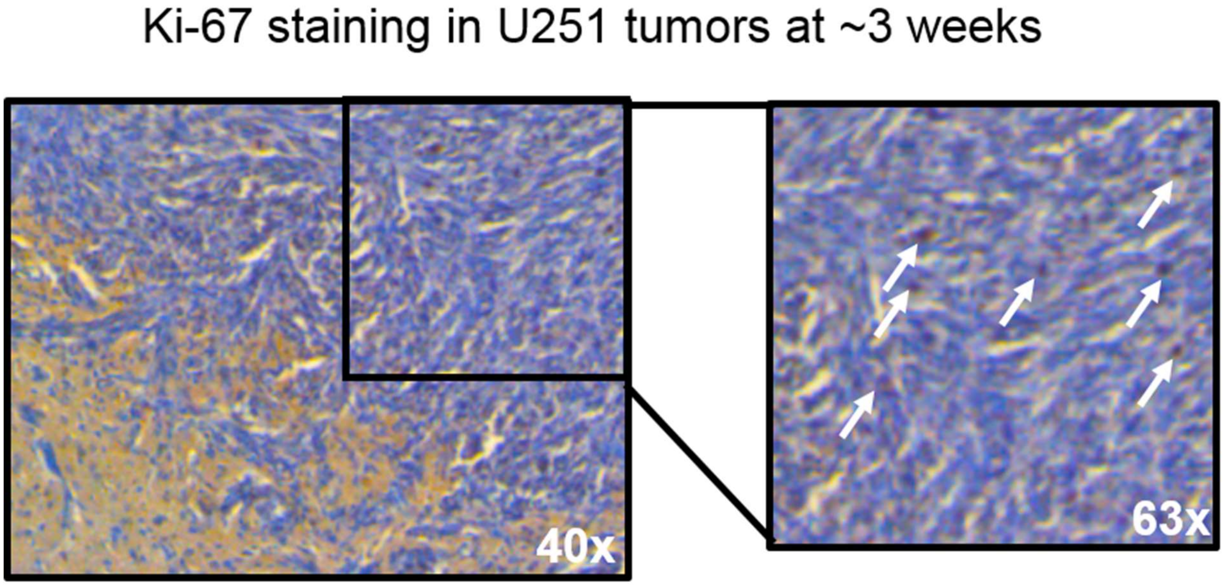

Supplementary Figure 2

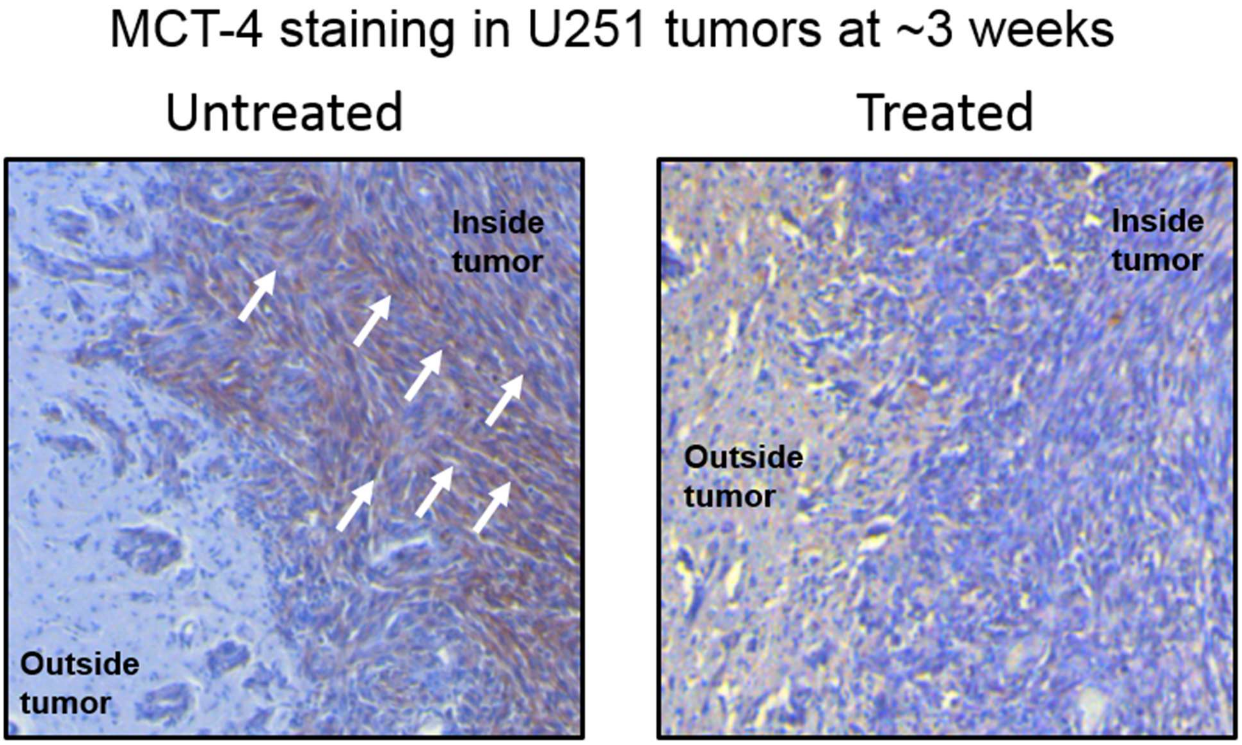

Supplementary Figure 1. Ki-67 staining of untreated U251 tumors in nude rat brain at ~3 weeks in 40x magnification. The enlarged area at 63x magnification with arrow marks pointing to positive DAB staining for Ki-67. Co-localization with hematoxylin (blue) is clearly visible.

Supplementary Figure 2. MCT-4 staining of U251 tumors in nude rat brain at ~3 weeks in 20x magnification in untreated and treated U251 tumors with arrow marks pointing to positive DAB staining for MCT-4.
